# IL-2-Mediated *In Vivo* Expansion of Regulatory T Cells Combined with CD154–CD40 Co-Stimulation Blockade but Not CTLA-4 Ig Prolongs Allograft Survival in Naive and Sensitized Mice

**DOI:** 10.3389/fimmu.2017.00421

**Published:** 2017-04-21

**Authors:** Lerisa Govender, Jean-Christophe Wyss, Rajesh Kumar, Manuel Pascual, Dela Golshayan

**Affiliations:** ^1^Transplantation Centre and Transplantation Immunopathology Laboratory, Service of Immunology and Allergy, Department of Medicine, Centre Hospitalier Universitaire Vaudois, University of Lausanne, Lausanne, Switzerland

**Keywords:** regulatory T cells, memory T cells, transplantation, tolerance, alloantibody

## Abstract

In recent years, regulatory T cells (Treg)-based immunotherapy has emerged as a promising strategy to promote operational tolerance after solid organ transplantation (SOT). However, a main hurdle for the therapeutic use of Treg in transplantation is their low frequency, particularly in non-lymphopenic hosts. We aimed to expand Treg directly *in vivo* and determine their efficacy in promoting donor-specific tolerance, using a stringent experimental model. Administration of the IL-2/JES6-1 immune complex at the time of transplantation resulted in significant expansion of donor-specific Treg, which suppressed alloreactive T cells. IL-2-mediated Treg expansion in combination with short-term CD154–CD40 co-stimulation blockade, but not CTLA-4 Ig or rapamycin, led to tolerance to MHC-mismatched skin grafts in non-lymphopenic mice, mainly by hindering alloreactive CD8^+^ effector T cells and the production of alloantibodies. Importantly, this treatment also allowed prolonged survival of allografts in the presence of either donor-specific or cross-reactive memory cells. However, late rejection occurred in sensitized hosts, partly mediated by activated B cells. Overall, these data illustrate the potential but also some important limitations of Treg-based therapy in clinical SOT as well as the importance of concomitant immunomodulatory strategies in particular in sensitized hosts.

## Introduction

Current immunosuppressive (IS) protocols are efficient in protecting against acute allograft rejection. However, the chronic administration of IS drugs is associated with toxicities and is often insufficient in preventing chronic allograft dysfunction, therefore hindering optimal patient and graft long-term outcome (1, 2). Due to their pleiotropic immune regulatory functions, the therapeutic potential of antigen-specific CD4^+^Foxp3^+^ regulatory T cells (Treg) has been highlighted in autoimmune diseases as well as in allogeneic hematopoietic stem cell and solid organ transplantation (SOT) (3–6). We and others have described targeted therapies based on *ex vivo* expansion of antigen-specific Treg that can, upon adoptive transfer into the recipient, control alloreactive effector T cells (Teff) and prevent acute and chronic allograft rejection (5, 7, 8). The constitutive low frequencies of thymic-derived Treg (tTreg) requires expansion of tTreg or induction of Treg from naïve conventional CD4^+^ T cells to achieve high Treg:Teff ratios for therapeutic efficacy after SOT, in particular in non-lymphopenic hosts (9–11). However, immunotherapeutic strategies based on *ex vivo* Treg production are challenging as they require highly selective purification and expansion methods in GMP facilities. Antigen-specificity is another issue implying the availability of donor-derived cells, as well as the *in vivo* stability and suppressive capacity of Treg after transfer (12–16).

An alternative approach would be the expansion of Treg directly *in vivo* in the presence of defined alloantigens. It has been shown that IL-2 signaling through the high-affinity IL-2R constitutively expressed on tTreg is essential for their homeostasis and IS function through the level of Foxp3 and CD25 expression (17). The clinical benefit of utilizing IL-2 to enhance Treg numbers has been reported in two recent publications (18, 19). However, a major problem with the therapeutic use of many cytokines is their short half-life in the circulation following administration (20). Furthermore, high-dose IL-2 may activate other cells expressing the IL-2R, such as memory CD8^+^ T cells and NK cells. Boyman et al. first described the use of an IL-2/JES6-1 complex (IL2c) that not only had a longer half-life than IL-2 alone but also could selectively expand Treg *in vivo* by sterically blocking IL-2/IL-2Rβ and IL-2/IL-2Rγ interactions and increasing the affinity of IL-2 to IL-2Rα^high^ Treg (21, 22). Additionally, treatment with a similar IL2c has been recently shown to increase the stability of Foxp3 expression (15).

In this study, we aimed to expand Treg directly *in vivo* using an IL2c and determine their suppressive function and efficacy in promoting donor-specific tolerance in a stringent MHC-mismatched skin transplantation (Tx) model. Since we predicted that the expansion of the Treg pool alone would not be sufficient to prevent rejection in non-lymphophenic hosts, we explored how IL2c treatment could be best combined with immunomodulatory drugs to control alloreactive T and B cells, in naïve as well as in pre-sensitized hosts. Our data demonstrate that IL2c administration combined with CD154–CD40, but not with CD28-B7 co-stimulation blockade or rapamycin (Rapa), could induce allograft tolerance in non-lymphopenic naive recipients. However, tolerance was not achieved in pre-sensitized hosts (harboring allospecific or cross-reactive memory), as late rejection occurred, partly mediated by activated B cells.

## Materials and Methods

### Mice

Wild-type C57BL/6 (B6, H2^b^), BALB/c (H2^d^), CBA (H2^k^), and B6xDBA2 F1 (B6D2, H2^b^xH2^d^) mice were purchased from Charles-River and Elevage Janvier. Unless specified, all experimental procedures were performed on 8- to 12-week-old sex- and age-matched female mice. All mice were maintained in the specific pathogen-free animal facilities of the CHUV.

### *In Vivo* Expansion of Treg

IL-2/anti-IL-2 complexes (IL2c) were prepared as previously described (21). In brief, 0.05 mg/kg recombinant mouse IL-2 was mixed with 0.25 mg/kg anti-IL-2 (clone JES6-1) and incubated at 37°C for 30 min. Mice were injected i.p. for three consecutive days.

### T-Cell Purification

Single-cell suspensions were obtained by passing spleens and lymph nodes (LN) through 70-µm cell-strainers. After erythrocytes lysis, cells were incubated with the following rat anti-mouse hybridoma culture supernatants: anti-MHCII (M5/114, TIB-120/ATCC; Manassas, VA, USA), anti-CD45R/B220 (RA3-3A1, TIB-146/ATCC), and anti-CD16/32 (2.4G2, HB-197/ATCC) followed by sheep anti-rat DynaBeads^®^ (Invitrogen) before separation in a magnetic field. CD4^+^ T cells were negatively selected using in addition anti-CD8 (YTS169; Therapeutic Immunology Group, Oxford, UK) in the purification cocktail. CD4*^+^*CD25^−^ and CD4^+^CD25^+^ subsets were selected using anti-CD25-biotinylated (clone 7D4; BD Biosciences) followed by Streptavidin-MicroBeads^®^ (MiltenyiBiotec, Germany), as described (5).

### Generation of Dendritic Cells (DC)

Fresh bone marrow cells flushed from femurs and tibias were passed through 70-µm cell-strainers. After erythrocytes lysis, BM cells were incubated with supernatants from YTS169, YTS191 (anti-CD4; Therapeutic Immunology Group, Oxford, UK), M5/114, and RA3-3A1, followed by sheep anti-rat Dynabeads^®^. After magnetic separation, cells were transferred to 24-well plates in the presence of 20 ng/ml mouse recombinant granulocyte/macrophage colony-stimulating factor (GM-CSF; R&D Systems). On days 2 and 4, medium containing small non-adherent cells was removed and replaced with fresh GM-CSF-containing medium. For maturation, 1 µg/ml LPS (*E. coli* 026:B6, Sigma) was added on day 6 for the final 12 h.

### Cell Cultures

Cell cultures were performed in RPMI-1640 (Sigma) supplemented with 100 IU/ml penicillin, 100 µg/ml streptomycin, 2mM l-glutamine, 0.01 M Hepes, 50 µM 2β-mercaptoethanol (Invitrogen), and 10% heat-inactivated fetal calf serum (FCS) (EuroClone, UK), incubated at 37°C in a humidified atmosphere with 5% CO_2_. Proliferation assays were performed in triplicates in 96-well plates. T cells (1 × 10^5^ cells/well) were stimulated with anti-CD3/CD28-coated beads (Dynabeads^®^ Mouse T-Activator; Invitrogen) at a 1:1 bead:cell ratio for 3 days. Alternatively, a mixed lymphocyte reaction (MLR) was performed for 6 days, using a 1:5 allogeneic DC:T ratio. For suppression assays, CD4^+^CD25^−^ (Tconv) were cultured alone or cocultured with CD4^+^CD25^+^ (Treg) T cells (1:1, 2:1, and 4:1 ratios) in the presence of allogeneic DC or polyclonal stimulation. Cell proliferation was measured by [^3^H]-thymidine incorporation (cpm) at 72 h of culture or by flow cytometry analysis of CFSE dilutions of labeled Tconv. The equation: [(cpm of Tconv alone − cpm of Tconv cultured with Treg)/(cpm of Tconv alone) × 100] was used to calculate the percentage of suppression.

### Skin Transplantation and *In Vivo* Treatments

All surgical procedures were performed under general anesthesia (xylazine/ketamine) and no pain-control medication was added afterward. Full-thickness tail skins from donors were grafted on beds prepared on the lateral flanks of female B6 recipients. Graft sites were protected under sterile gauze and a plaster, removed at day 10. Grafts were observed daily afterward and were considered rejected when no viable skin remained. Mice were injected i.p. with 10 mg/kg cyclosporine A (CsA) (Sandimmun, Novartis Pharma Schweiz) from day 0 to 10; 25 mg/kg anti-CD40L (anti-CD154, clone MR1; BioXcell, West Lebanon, NH, USA) on days 0, 2, and 4; 12.5 mg/kg CTLA-4 Ig on days 0, 2, 4, and 6 (Belatacept; Bristol-Myers Squibb); 10 mg/kg anti-LFA-1 (anti-M17/4, BioXcell) on days 0, 2, and 4; or received 1 mg/kg Rapa (Rapamune; Pfizer AG) by oral gavage from day 1 to 10 post Tx. In some experiments, 3 mg/kg Rapa (Calbiochem) was given i.p. from day 0 to 5. To obtain immunization, recipient female B6 mice were injected s.cut with 5 × 10^5^ DC from B6D2 or male B6 donors, or 0.5 mg/kg of azide/endotoxin-free anti-CD3 (145-2C11 eBioscience) 8–10 weeks prior to Tx.

### Antibodies and Flow Cytometry

The following anti-mouse fluorochrome-conjugated mAbs and their respective isotype controls were used: CD3 (145-2C11), CD4 (clone RM4-5), CD8 (53-6.7), CD44 (IM7), CD49d (DX5), CD62L (MEL-14), B220 (RA3-6B2), IL-2 (JES6-5H4), IFN-γ (XMG1.2), IL-10 (JES5-16E3), IL-17 (TC11-18H10), MHC class II (I-A/I-E), PD-1 (CD279), all purchased from BD Biosciences; Helios (22F6) from Biolegend; and CD25 (PC61) and Foxp3 (FJK-16s) from eBioscience. For the detection of cytokines, cells from spleen and LN were resuspended at approximately 4 × 10^6^ cells/ml in RPMI-10% FCS, restimulated with 50 ng/ml phorbol 12-myristate 13-acetate (PMA) and 0.5 µg/ml ionomycin for 5 h, in the presence of 10 µg/ml Brefeldin A (Sigma). Cells were then harvested, surface stained, fixed for 10 min in BD FACS Lysing Solution (BD Biosciences), and washed and permeabilized with PermBuffer (eBioscience) before intracellular staining. Intracellular Foxp3 staining was performed using a Foxp3 staining kit (eBioscience). The staining of antigen-specific CD4^+^ T cells was carried out using the MHC class II I-Ab HY (116-130) NAGFNSNRANSSRSS tetramer (TCMetrix, Epalinges, Switzerland). Flow cytometry acquisition was done on FACS-Calibur™ using CellQuest™ (BectonDickinson) and data analyzed using FlowJo 9.5.3 software (Treestar).

### Histology

Skin grafts were fixed in 4% buffered formalin and embedded in paraffin. Sections (6 µm) were stained with H&E, according to standard protocols. For immunohistochemistry, sections were baked at 55°C for 20 min, cooled, deparaffinized, and rehydrated through graded alcohols to water. For antigen retrieval, slides were placed in pre-heated (95–100°C) Tris–EDTA buffer (10 mM Tris base, 1 mM EDTA, 0.025% Tween 20, pH 9.0) and microwaved for 20′ at 240 W. Endogenous peroxidase and biotin were blocked by 10′ incubation in 3% hydrogen-peroxide and Biotin Blocking System (Dako), respectively. After 30′ blocking in PBS-4% BSA-0.05% Tween 20, the slides were incubated with biotinylated anti-Foxp3 (clone FJK-16S, eBioscience), followed by Streptavidin/HRP (Dako). Slides were washed, incubated with liquid diaminobenzidine-tetrahydrochloride plus substrate (DakoCytomation) for 15′, rinsed with water, and counterstained with hematoxylin. All images were captured with a Nikon Eclipse E800 microscope and digital DXM1200 camera.

### Detection of Donor-Specific IgG

Serum was collected from control non-transplanted, rejecting, and tolerant (day 100 post Tx) B6 recipient mice at the time of sacrifice and heat inactivated at 56°C for 30 min. Thymocytes from donor (B6D2), syngeneic, and third party (CBA) strains were collected and passed through 70-µm cell-strainers to obtain single-cell suspensions. Heat-inactivated non-diluted serum was incubated with 1 × 10^6^ thymocytes at 37°C for 30 min. The binding of serum IgG to thymocytes was detected by flow cytometry using fluorochrome-conjugated polyclonal anti-IgG (Poly4060; Biolegend). A negative control of B6D2 thymocytes stained with IgG without prior incubation with serum was included. Alloantibody levels were quantified by analyzing the mean fluorescence intensity (MFI) of bound IgG Abs.

### Statistical Analysis

All experiments were repeated at least twice. Unpaired two-tailed Students *t*-test (comparison between two groups) and one-way ANOVA with Tukey’s post-test for multiple comparisons were used to calculate significance levels between treatment groups (GraphPad Prism version 6, CA, USA). Graft survival (median survival time, MST) was analyzed by Kaplan–Meier curves and the log-rank test. *P* values <0.05 were considered significant (*P < 0.05, **P < 0.01, and ***P < 0.001).

## Results

### The IL-2/JES6-1 Complex (IL2c) Expands Functional Treg *In Vivo*

We first addressed how we could best take advantage of directly expanding Treg *in vivo* and, therefore, determined kinetics of immune cell-subsets following IL2c administration in the blood on days 3, 5, 7, and 10 after treatment as compared to PBS-treated controls (Figure S1 in Supplementary Material). In the absence of immune activation, we observed a selective and significant increase in CD4^+^CD25^+^CD44^+^Foxp3^+^ T cells frequencies and numbers (3.3 vs.14.9% and 18 vs. 82 cells/100 µl blood; in PBS and IL2c groups, respectively), that peaked on day 5, persisted until day 7, and returned to baseline by day 10 (Figure S1A–G in Supplementary Material). At day 10, further analysis of the immune cell subsets in peripheral lymphoid organs showed that, in the absence of immune activation, higher frequencies of CD4^+^Foxp3^+^ T cells only persisted in the spleen (Figure S1H–N in Supplementary Material).

**Figure 1:**
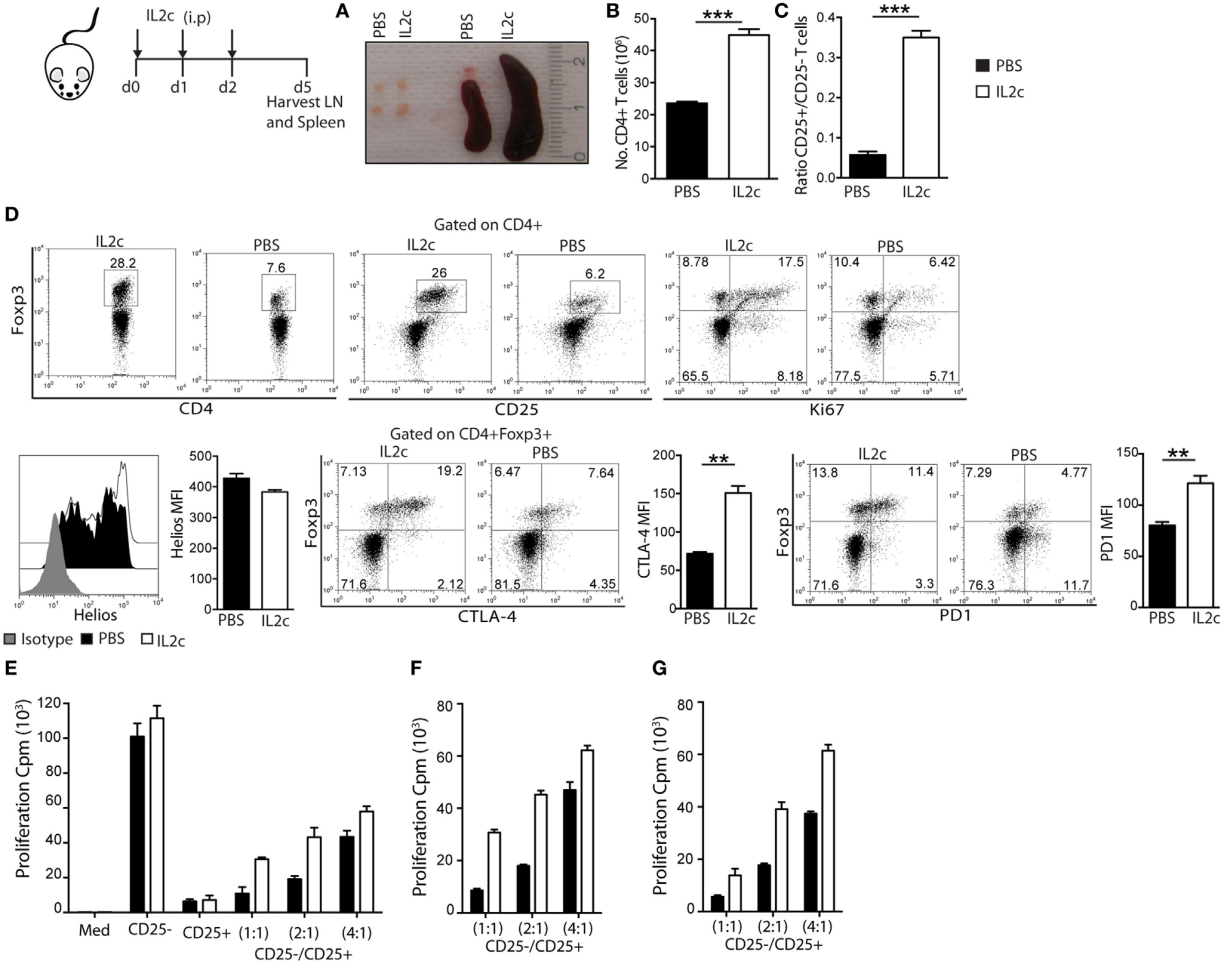
**Phenotype and suppressive function of regulatory T cells expanded *in vivo* with IL2c**. BALB/c mice received IL2c (open bars) or PBS (solid bars) treatment i.p. on days 0, 1, and 2. On day 5, spleen and peripheral axillary and inguinal lymph nodes (LN) were harvested. **(A)** Peripheral LN and spleen in PBS and IL2c-treated mice. **(B)** Total number of CD4^+^ T cells in pooled spleen and LN after T-cell purification. **(C)** Ratio of CD25^+^/CD25^−^ within purified CD4^+^ T cells. **(D)** Representative plots and summary data of LN cells for the expression of Foxp3, CD25, Ki67, Helios, CTLA-4, and PD-1 (gated on CD4^+^ or CD4^+^Foxp3^+^). **(E)** CD4^+^CD25^−^ T cells (1 × 10^5^) were cultured alone or cocultured with CD4^+^CD25^+^ T cells (cell ratios of 1:1, 2:1, or 4:1) and anti-CD3/CD28 beads. Alternatively, **(F)** CD25^−^ subset isolated from PBS-treated cultured with CD25^+^ from PBS or IL2c-treated mice; or **(G)** CD25^−^ from IL2c-treated with CD25^+^ from PBS or IL2c-treated mice; in the presence of anti-CD3/CD28 beads. All conditions were plated in triplicates and the data are representative of three independent experiments. Dot-plots show the percentage of subsets within regions/quadrants, bar graphs represent mean ± SEM. *n* = 3–4 mice/group (*P < 0.05, **P < 0.01, and ***P < 0.001).

We next investigated whether these *in vivo*-expanded Treg retained their phenotype and suppressive function. BALB/c mice were treated with either IL2c or control PBS for three consecutive days, followed by harvest of spleen and peripheral axillary and inguinal LN on day 5 (corresponding to the peak rate of expansion) for cell counts and characterization. Enlarged lymphoid organs and increased total cell counts were observed in IL2c as compared to PBS-treated mice, with a sevenfold higher CD25^+^/CD25^−^ ratio within the CD4^+^ population in treated mice (Figures 1A–C). We confirmed a selective expansion of CD4^+^CD25^+^Foxp3^+^ T cells by day 5 of IL2c treatment, as determined by Ki67 expression by flow cytometry (Figure 1D). Foxp3^+^ T cells from control PBS-treated mice or after *in vivo* expansion had a similar expression of Helios, suggesting preferential expansion of tTreg rather than peripheral induction of Treg upon IL2c treatment in steady-state conditions (23). Additionally, the surface expression of the inhibitory molecules PD-1 and CTLA-4 was significantly increased in IL2c-expanded Treg. To determine the suppressive function of IL2c-expanded Treg, CD4^+^CD25^+^ (Treg) and CD4^+^CD25^−^ (Tconv) T-cell subsets were purified from both groups for *in vitro* cocultures. While IL2c-expanded Treg suppressed the proliferation of Tconv in a dose-dependent manner in response to polyclonal activation, they tended to be less suppressive than control Treg on a cell-per-cell basis (Figures 1E–G). However, control Treg (purified from PBS-treated mice) could similarly suppress Tconv from both groups, ruling out increased responsiveness of Tconv after *in vivo* exposure to IL2c. Overall, these results demonstrated that treatment with the IL2c selectively expanded functional Treg *in vivo*.

### IL2c-Expanded Treg Promote Prolonged Survival of MHC-Mismatched Skin Allografts

**Figure 2:**
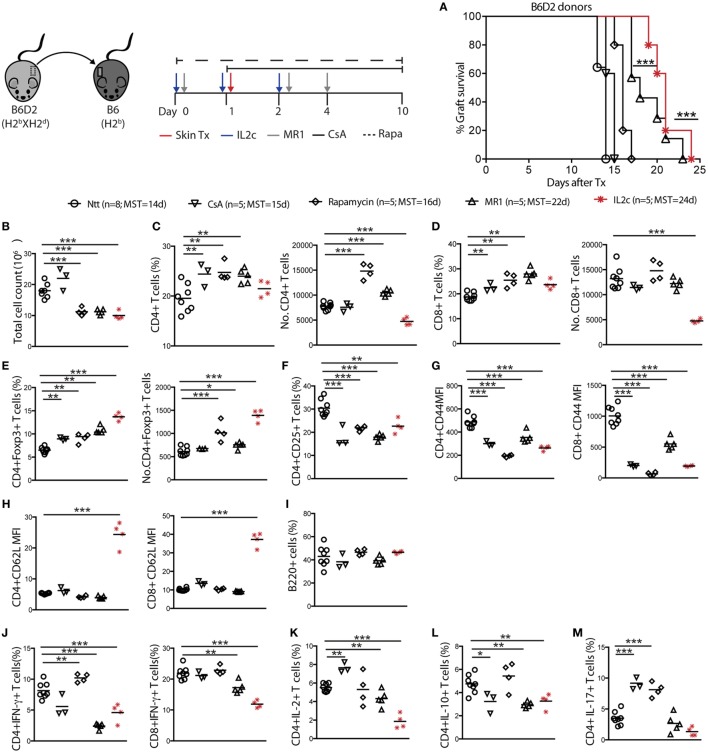
**IL2c-expanded Treg prolong MHC-mismatched skin grafts survival in non-lymphopenic mice**. B6 mice were treated with IL2c or drugs and underwent B6D2 skin grafts. **(A)** Graft survival. Mice were sacrificed at rejection to analyze the spleen and graft draining lymph nodes (LN) (not shown for spleen). **(B)** Total cell counts in the graft draining LN. Frequency and numbers of **(C)** CD4^+^; **(D)** CD8^+^; **(E)** CD4^+^Foxp3^+^; and **(F)** CD4^+^CD25^+^ T cells. **(G,H)** MFI of CD44 and CD62L expression on CD4^+^ and CD8^+^ T cells. Frequency of **(I)** B220^+^ B cells; **(J)** IFN-γ^+^ CD4^+^; and CD8^+^ T cells; **(K–M)** IL-2^+^, IL-10^+^, and IL-17^+^ CD4^+^ T cells. MFI, mean fluorescence intensity; MST, median survival time. *n* = 5–8 mice/group (*P < 0.05, **P < 0.01, and ***P < 0.001).

We reasoned that with the *in vivo* expansion of Treg at the time of Tx, the strong alloreactive T-cell pool could be controlled, resulting in prolonged allograft survival. Wild-type non-lymphopenic C57BL/6 (B6) recipient mice were grafted with B6xDBA2 F1 (B6D2) donors skins and either received no drugs (controls), intraperitoneal injection (i.p.) of IL2c starting the day prior to Tx, or other known IS drugs. Recipients treated with either CsA or Rapa rejected their allografts in a similar tempo as compared to the control non-treated group (MST = 15, 16, and 14 days; in CsA, Rapa, and control groups, respectively). Treatment with either IL2c or anti-CD154 mAb (MR1) alone resulted in significant prolongation of graft survival in this MHC-mismatched skin Tx setting (MST = 24, ***P < 0.0001 and 22 days, ***P < 0.0001, respectively) (Figure 2A).

Cell suspensions were prepared from spleen and graft draining LN (dLN) of recipient mice at rejection to analyze alloreactive T-cell responses (dLN data only shown). As compared to controls and CsA-treated mice, Rapa, MR1, and IL2c treatments resulted in significant higher frequencies of CD4^+^Foxp3^+^ T cells, with IL2c-treated mice displaying the highest numbers (Figures 2B–E). All treatments resulted in lower CD4^+^ and CD8^+^ T-cell activation as judged by the surface expression of CD25 and CD44 (Figures 2F,G). Interestingly, the LN homing receptor L-selectin (CD62L) remained significantly upregulated on CD4^+^ and CD8^+^ T cells after IL2c treatment (Figure 2H), suggesting that this treatment and/or the presence of expanded Treg prevented effector/memory differentiation. No differences in the frequency of B220^+^ B cells were observed among groups in the graft dLN at rejection (Figure 2I). Finally, in contrast to mice that rejected their allograft at an earlier time-point, MR1 and IL2c-treated mice had significantly lower frequencies of CD4^+^IFN-γ^+^, CD8^+^IFN-γ^+^, CD4^+^IL-2^+^, as well as CD4^+^IL-17^+^ Teff, however without favoring IL-10-producing CD4^+^ T cells (Figures 2J–M). Overall, IL2c treatment allowed the expansion of the Treg pool even after Tx and immune activation, which may have to some extent controlled the activation and proliferation of CD4^+^ and CD8^+^ Teff in response to the allograft, resulting in prolonged graft survival.

### IL2c Combined with Anti-CD154 Promotes MHC-Mismatched Skin Graft Tolerance in Immunocompetent Mice

Increasing the number of Treg at the time of Tx proved insufficient to promote graft tolerance, suggesting that additional immunomodulatory therapies would be necessary to regulate the strong early direct pathway alloresponse in our model. Therefore, we investigated whether Rapa or MR1 had an effect on the *in vivo* expansion of Treg when administered in combination with IL2c in steady-state conditions, i.e., in the absence of an immune response. We chose not to use CsA in further experiments as by inhibiting TCR downstream calcineurin-dependent signaling, therefore IL-2 production, CsA was shown to compromise Treg homeostasis and function (24). As compared to PBS-treated mice, there was an enlargement of the LN and spleen (Figure S2A in Supplementary Material), as well as increased total cell numbers (data not shown) in mice treated either with IL2c alone or in combination with Rapa or MR1. This was mainly due to an increase in the frequency of CD4^+^Foxp3^+^ T cells, with proportionally decreased frequencies of CD8^+^ T cells in the LN and spleen (Figure S2B–I in Supplementary Material). Therefore, therapies combining IL2c with Rapa or MR1 allowed preferential expansion of Treg *in vivo*.

**Figure 3:**
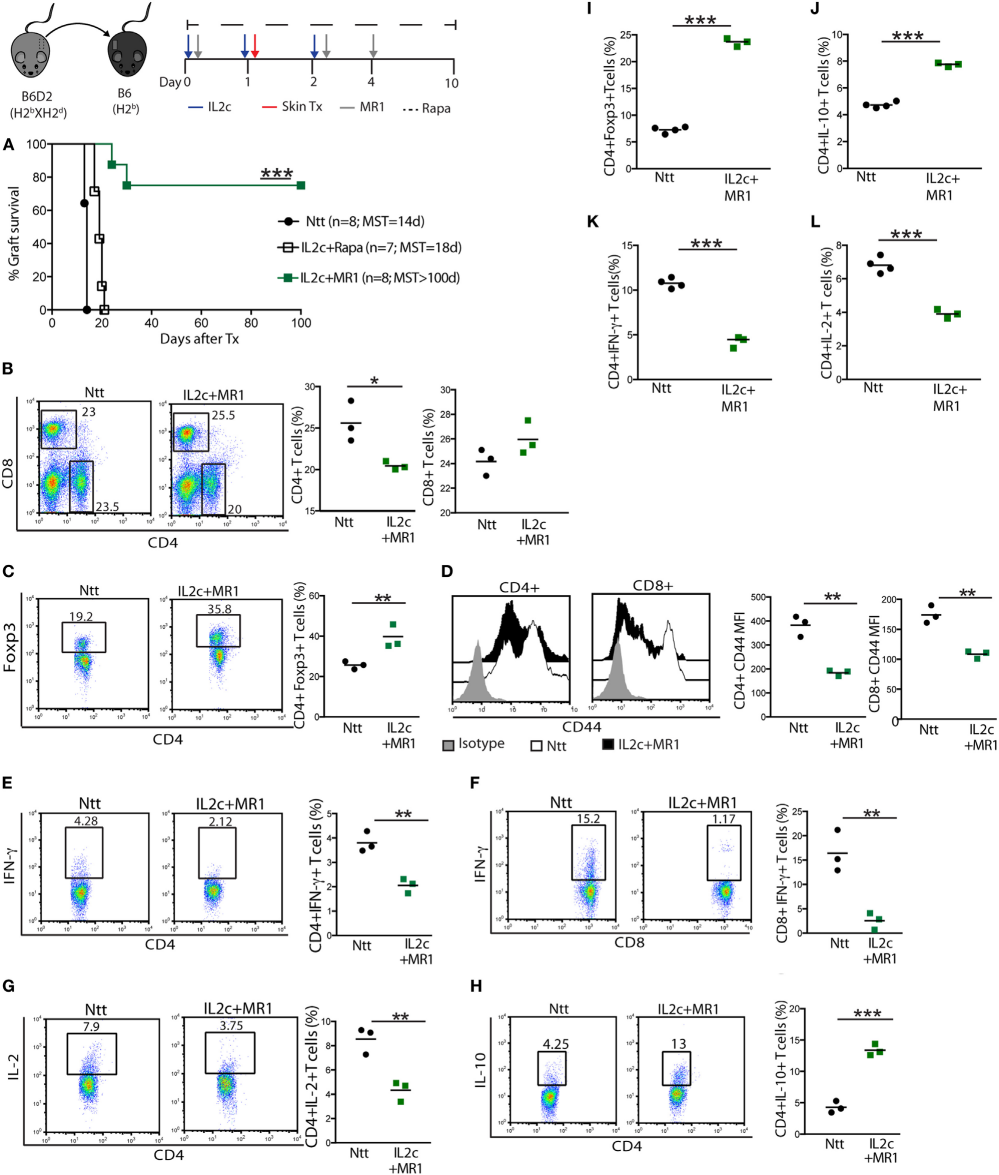
**The combination of IL2c with MR1, but not rapamycin (Rapa), promotes tolerance to MHC-mismatched skin grafts in immunocompetent mice**. B6 mice received IL2c with MR1, Rapa, or were left untreated (Ntt), and underwent B6D2 skin grafts. **(A)** Graft survival. Mice from control Ntt and IL2c + MR1 groups were sacrificed on day 7 to monitor early immune effectors in the graft draining lymph nodes. Frequency of **(B)** CD4^+^ and CD8^+^, **(C)** CD4^+^Foxp3^+^ T cells. **(D)** CD44 expression on CD4^+^ and CD8^+^ T cells. Frequency of **(E–H)** CD4^+^IFN-γ^+^, CD8^+^IFN-γ^+^, CD4^+^IL-2^+^, and CD4^+^IL-10^+^ T cells. Frequency of **(I)** CD4^+^Foxp3^+^ T cells; **(J–L)** IL-10^+^, IFN-γ^+^ and IL-2^+^ CD4^+^ T cells; in rejecting (day 14) and tolerant mice (day 100). MST, median survival time. *n* = 3–5 mice/group (*P < 0.05, **P < 0.01, and ***P < 0.001).

Thus, we hypothesized that Rapa or MR1 given in combination with IL2c at the time of Tx may control the alloreactive T-cell pool and proportionally increase Treg. The graft survival curves presented in Figure 3A clearly demonstrate that IL2c in combination with CD154–CD40 co-stimulation blockade, but not with Rapa, could promote tolerance to MHC class I-and II-mismatched skin grafts [MST > 100, 18, and 14 days; in IL2c + MR1, IL2c + Rapa, and control non-treated (Ntt) groups, respectively] in immunocompetent recipients. Allografts from tolerant mice were macroscopically healthy at day 100 with no retraction or necrosis and displayed hair growth. To determine the mechanisms leading to the induction and maintenance of tolerance, we analyzed and compared alloreactive T-cell responses in the dLN (Figure 3) and spleen (data not shown) of control and IL2c + MR1-treated mice at day 7 as well as day 100 after Tx. Early after Tx, the presence of an immunomodulatory drug allowed slight reduction of the frequency of alloreactive CD4^+^ T cells, with an increase in the proportion of Foxp3^+^ cells (40 and 23%, in IL2c + MR1 and control groups, respectively) (Figures 3B,C). There was in addition a marked decrease in the population of activated CD4^+^ and CD8^+^ T cells, as well as of CD4^+^IFN-γ^+^, CD8^+^IFN-γ^+^, and CD4^+^IL-2^+^ Teff together with increased frequencies of CD4^+^IL-10^+^ T cells (Figures 3D–H). Even at the late time-point of 100 days, IL2c + MR1-treated tolerant mice displayed an immunoregulatory phenotype with sustained higher frequencies of CD4^+^Foxp3^+^ T cells and CD4^+^IL-10^+^ T cells compared to control non-treated mice that rejected their allografts after Tx (Figures 3I,J). IL2c + MR1 also promoted maintained significant reduction in CD4^+^IFN-γ^+^ and CD4^+^IL-2^+^ Teff (Figures 3K,L). Hence, the expansion of Treg at the time of Tx in combination with short-term CD154–CD40 co-stimulation blockade shifted the alloreactive T-cell pool toward regulation, favoring Tx tolerance.

### The Combination of IL2c and Anti-CD154 Efficiently Regulates Donor-Specific Cellular and Humoral Alloimmune Responses

**Figure 4:**
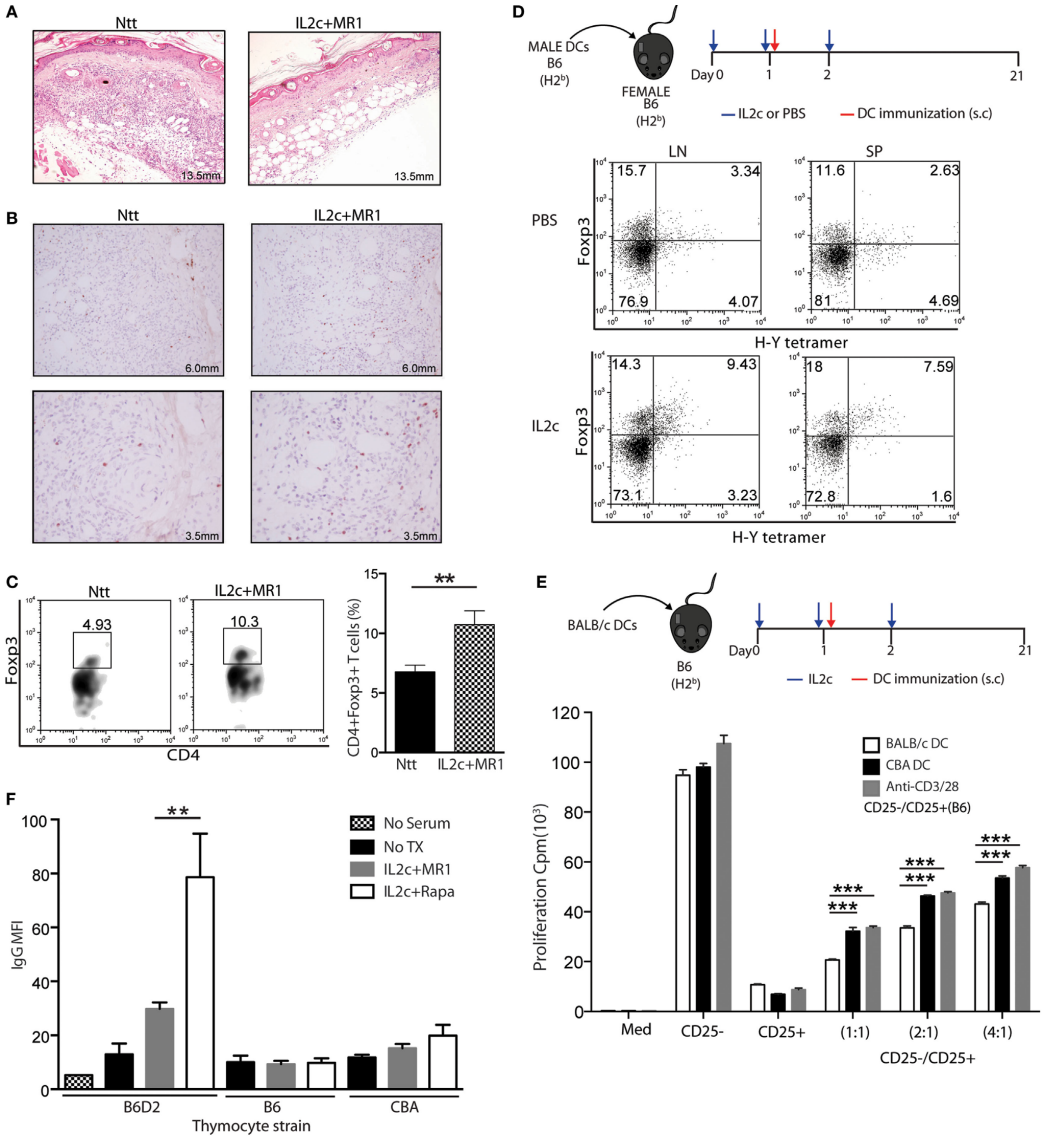
**IL2c in combination with MR1 regulates donor-specific alloimmune responses**. **(A–C)** Skin grafts from control non-treated (Ntt) and IL2c + MR1-treated tolerant mice were analyzed at day 7. **(A)** H&E staining. **(B)** Foxp3 immuno-staining. **(C)** Frequency of CD4^+^Foxp3^+^ T cells. **(D)** Frequency of I-Ab H-Y tetramer^+^ CD4^+^Foxp3^+^ T cells in lymph nodes and spleen from PBS- or IL2c-treated female B6 mice, immunized with male B6 dendritic cells (DC). **(E)** *In vitro* rechallenge assay. CD25^−^ and CD25^+^ CD4^+^ T-cell subsets were isolated from IL2c-treated B6 mice at day 21 after immunization with 5 × 10^5^ BALB/c DC and cocultured at different ratios in the presence of allogeneic or third party CBA DC, or polyclonal stimulation. **(F)** Presence of donor-specific IgG antibodies in the serum of non-transplanted, graft rejected (IL2c + rapamycin), or tolerant (IL2c + MR1) mice. Donor (B6D2), syngeneic (B6), or third party (CBA) strains thymocytes were used. Representative histology and flow cytometry stainings are shown from one out of 3–4 mice/group. All *in vitro* cultures were done in triplicates and are representative of two independent experiments. Bar graphs indicate mean ± SEM (*P < 0.05, **P < 0.01, and ***P < 0.001).

Next, we analyzed the homing properties of the Treg expanded *in vivo* at the time of an allogeneic Tx. On day 7, the histological integrity of grafted skins was preserved in IL2c + MR1-treated mice (Figure 4A) in contrast to control grafts that showed marked lymphocytic infiltration and tissue destruction. A greater number of Foxp3^+^ cells could be identified in the grafted skins of IL2c + MR1-treated mice by immunohistochemistry (Figure 4B), as also confirmed by flow cytometry analysis of digested grafts showing a significant twofold increase in Foxp3^+^ cells in comparison to control mice (Figure 4C). These results indicated that expanded Treg migrated to the allograft to prevent tissue damage by alloreactive Teff.

The importance of antigen specificity in the effectiveness of Treg function in the setting of SOT has been previously demonstrated (5, 25, 26). Therefore, we investigated whether Treg expanded directly *in vivo* at the time of antigen exposure would gain antigen-specificity. Female B6 mice were injected subcutaneously (s.cut) with male B6 DC on day 1 of IL2c treatment. On day 21 post immunization, male antigen-specific CD4^+^ T cells were detected in secondary lymphoid organs using a MHC class II tetramer specific for the immunodominant minor *DbY*-encoded H-Y antigen. As compared to PBS-treated immunized mice, IL2c treatment allowed expansion of CD4^+^Foxp3^+^ Treg with enrichment of the H-Y^+^ fraction (Figure 4D), preferentially at the injection site dLN. To determine whether IL2c-expanded Treg could suppress alloreactive T cells in an antigen-specific manner, B6 mice were immunized with BALB/c DC on day 1 of IL2c treatment. CD4^+^CD25^+^ and CD4^+^CD25^−^ T cells were selected from these mice to perform an *in vitro* suppression assay in the presence of DC from either BALB/c or third-party CBA mice. Treg expanded in the presence of BALB/c DC were significantly more efficient at suppressing the proliferation of Tconv stimulated with BALB/c in comparison to CBA DC or polyclonally (Figure 4E). Altogether, these data suggested that the expansion of Treg in the presence of donor antigens allowed enriching the pool in antigen-specific Treg, thus increasing their suppressive capacity specifically toward donor antigens compared to polyclonal natural Treg.

Donor-specific alloantibodies (DSA) have been implicated in promoting both acute and chronic rejection (27, 28). We monitored the appearance of DSA in control and treated mice by flow cytometry (29) and observed significant lower MFIs of IgG in the sera of tolerant IL2c + MR1-treated compared to rejecting IL2c + Rapa-treated mice (Figure 4F). Control serum from non-grafted mice and recipient serum incubated with syngeneic B6 or third-party CBA thymocytes had background levels of IgG. This indicated that IL2c + MR1 therapy controlled alloreactive B-cell activation and the production of alloantibodies, contributing to long-term graft acceptance. As tolerance was not achieved with IL2c alone or combined with Rapa, these results suggested that the CD154–CD40 pathway plays an important role in antibody-mediated chronic rejection.

### CD28-B7 Co-Stimulation Blockade Allows *In Vivo* Expansion of Treg, without Promoting Allograft Tolerance

Next, we investigated whether blocking the CD28-B7 co-stimulation pathway using CTLA-4 Ig, a fusion protein currently used as maintenance therapy in kidney transplant recipients, in combination with IL2c would also promote allograft survival in our model. CTLA-4 Ig also allowed significant expansion of Treg *in vivo*, similar to IL2c + Rapa or IL2c + MR1 treatment. Indeed, increased frequencies of CD4^+^Foxp3^+^ Treg were present in the enlarged LN and spleen of mice treated with IL2c in combination with CTLA-4 Ig, with proportionally decreased frequencies of CD8^+^ T cells (Figure S2 in Supplementary Material).

**Figure 5:**
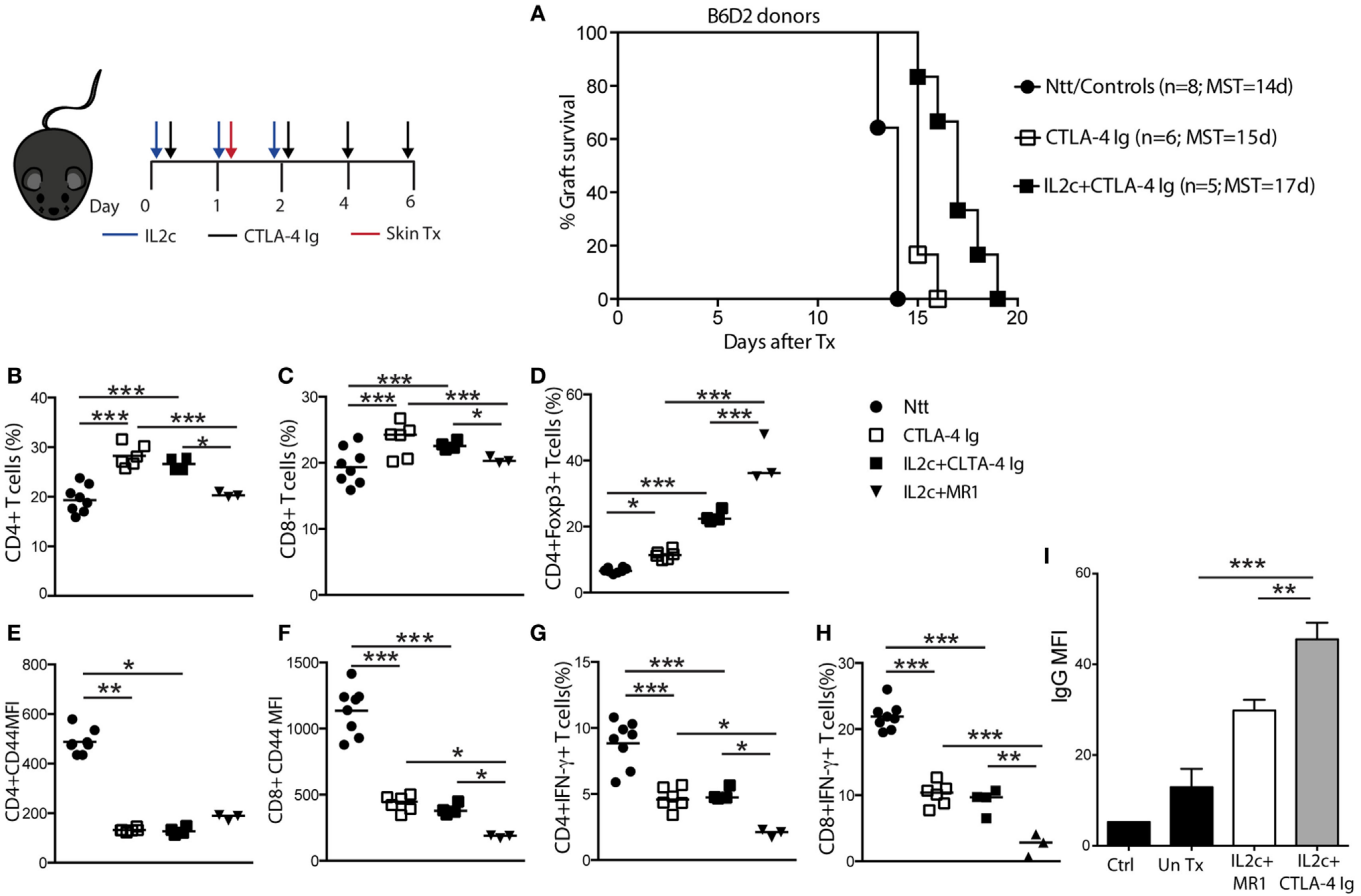
**Effect of CTLA-4 Ig co-stimulation blockade on IL2c-mediated Treg expansion and skin allograft survival**. B6 mice received no treatment (Ntt), CTLA-4 Ig alone, or in combination with IL2c and underwent B6D2 skin grafts. Mice were sacrificed at rejection to analyze graft draining lymph nodes cells in comparison to tolerant IL2c + MR1-treated mice, at a similar time-point after transplantation. **(A)** Skin graft survival. Frequency of **(B)** CD4^+^; **(C)** CD8^+^, and **(D)** CD4^+^Foxp3^+^ T cells. **(E,F)** Mean fluorescence intensity (MFI) of CD44 expression on CD4^+^ and CD8^+^ T cells, respectively. **(G,H)** Frequency of IFN-γ^+^ CD4^+^ and CD8^+^ T cells, respectively. **(I)** Detection of donor-specific IgG antibodies in the serum of IL2c + CTLA-4 Ig and IL2c + MR1-treated mice. *n* = 3–8 mice/group (*P < 0.05, **P < 0.01, and ***P < 0.001).

When B6 mice were grafted with B6D2 donor skins, treatment with either CTLA-4 Ig alone or IL2c + CTLA-4 Ig marginally prolonged allograft survival (MST = 15 and 17 days, respectively) (Figure 5A). Alloreactive T- and B-cell responses of IL2c + CTLA-4 Ig-treated recipients were compared to that of IL2c + MR1-treated mice at the same time-point (day 7 post Tx). Although the addition of CTLA-4 Ig allowed to some extent the expansion of Treg and controlled the activation of CD4^+^ and CD8^+^ T cells and IFN-γ production when compared to controls, it was not as effective as IL2c + MR1 in particular regarding CD8^+^ T-cell activation and effector function (Figures 5B–H). Furthermore, IL2c + CTLA-4 Ig could not efficiently prevent alloantibody production (Figure 5I). Taken together, these findings supported that in combination with IL2c, targeting the CD28–B7 pathway was insufficient to dampen the alloresponse across MHC barriers in non-lymphopenic recipients, in particular due to inadequate control of CD8^+^ Teff and the production of DSA.

### Treg Function in Response to Alloantigens Is Better Preserved under CD154–CD40 Blockade

**Figure 6:**
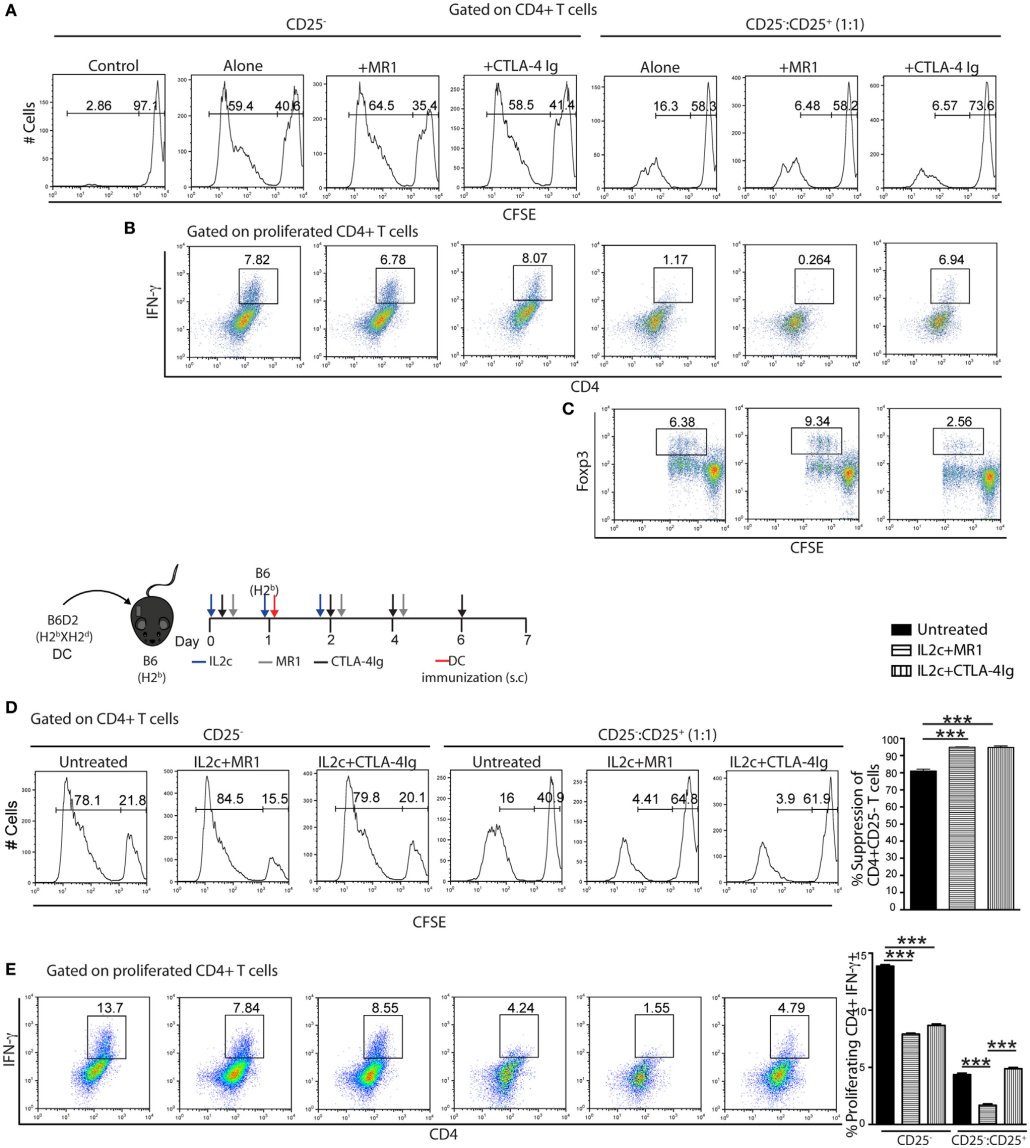
**Suppressive function of IL2c-expanded Treg in the presence of co-stimulation blockade**. **(A–C)** CD4^+^CD25^−^ (CD25^−^) and CD4^+^CD25^+^ (CD25^+^) T cells were isolated from naïve B6 mice. CFSE-labeled CD25^−^ cells (1 × 10^5^) were cultured alone or 1:1 with unlabeled CD25^+^ in the presence of 0.2 × 10^5^ allogeneic B6D2 dendritic cells (DC) and various drugs for 6 days. **(D,E)** B6 mice were immunized with 5 × 10^5^ allogeneic B6D2 DC and either left untreated or received IL2c + MR1 or IL2c + CTLA-4 Ig. On day 7 after immunization, CD4^+^ T-cell subsets were isolated from pooled lymphoid organs and the CFSE-labeled CD25^−^ subset was cocultured with CD25^+^ and B6D2 DC for 6 days before assessment by flow cytometry. **(A,D)** CFSE dilutions. **(B,E)** Frequency of IFN-γ^+^CD4^+^ proliferating T cells. **(C)** Foxp3 expression by CD4^+^ proliferating T cells. All *in vitro* conditions were plated in triplicates and data are shown from one out of two representative experiments. Bar graphs indicate mean ± SEM (***P < 0.001).

The competitive interaction of CTLA-4 constitutively expressed on Treg with B7.1/2 ligands is one mechanism used by Treg to regulate APC and promote the production of indoleamine 2,3-dioxygenase (IDO), a potent immunoregulatory molecule (30). Therefore, we investigated whether CTLA-4 Ig may interfere with Treg-mediated immune regulation in our setting. CD4^+^ T-cell subsets were purified from non-manipulated B6 mice and CD4^+^CD25^+^ Treg were cocultured with CFSE-labeled CD4^+^CD25^−^ Tconv for 6 days, in the presence of allogeneic B6D2 DC and either MR1 or CTLA-4 Ig. In a primary MLR, blocking either the CD154–CD40 or CD28–B7 pathway had no effect on Tconv or on the suppressive capacity of Treg (Figure 6A). However, as compared to CTLA-4 Ig, the addition of MR1 in the CD25^−^:CD25^+^ cocultures resulted in slightly more efficacious inhibition of CD4^+^IFN-γ^+^ Teff and allowed significant induction and expansion of CD4^+^Foxp3^+^ Treg in the presence of allogeneic DC (Figures 6B,C).

We further evaluated the potential of combining IL2c with either MR1 or CTLA-4 Ig in dampening antigen-specific Teff responses upon rechallenge. B6 mice were immunized with B6D2 DC while receiving either no treatment (untreated), IL2c + MR1, or IL2c + CTLA-4 Ig. Tconv and Treg were isolated from the different groups on day 7 and rechallenge MLR were performed in the presence of B6D2 DC. Compared to naïve Treg (isolated from non-immunized mice, histogram as for Figure 6A), *in vivo* antigen-experienced Treg were more suppressive. Additionally, Treg expanded in the presence of MR1 or CTLA-4 Ig had enhanced suppressive capacity compared to Treg from the untreated group (Figure 6D). As compared to Tconv purified from immunized untreated mice, the *in vitro* proliferation of Tconv was not modified by any previous *in vivo* drug exposure; however, *in vivo* exposure to both MR1 and CTLA-4 Ig significantly diminished the frequency of allospecific CD4^+^IFN-γ^+^ Teff. Moreover, in cocultures only previous exposure to MR1 further increased suppression by *in vivo*-expanded Treg (Figure 6E). Thus, in the setting of an allospecific recall response, treatment with MR1 not only enhanced Treg suppressive capacity but also prevented antigen-specific Teff differentiation.

### IL2c Combined with Anti-CD154 Prolongs Allograft Survival in the Presence of Preexisting Memory T Cells

**Figure 7:**
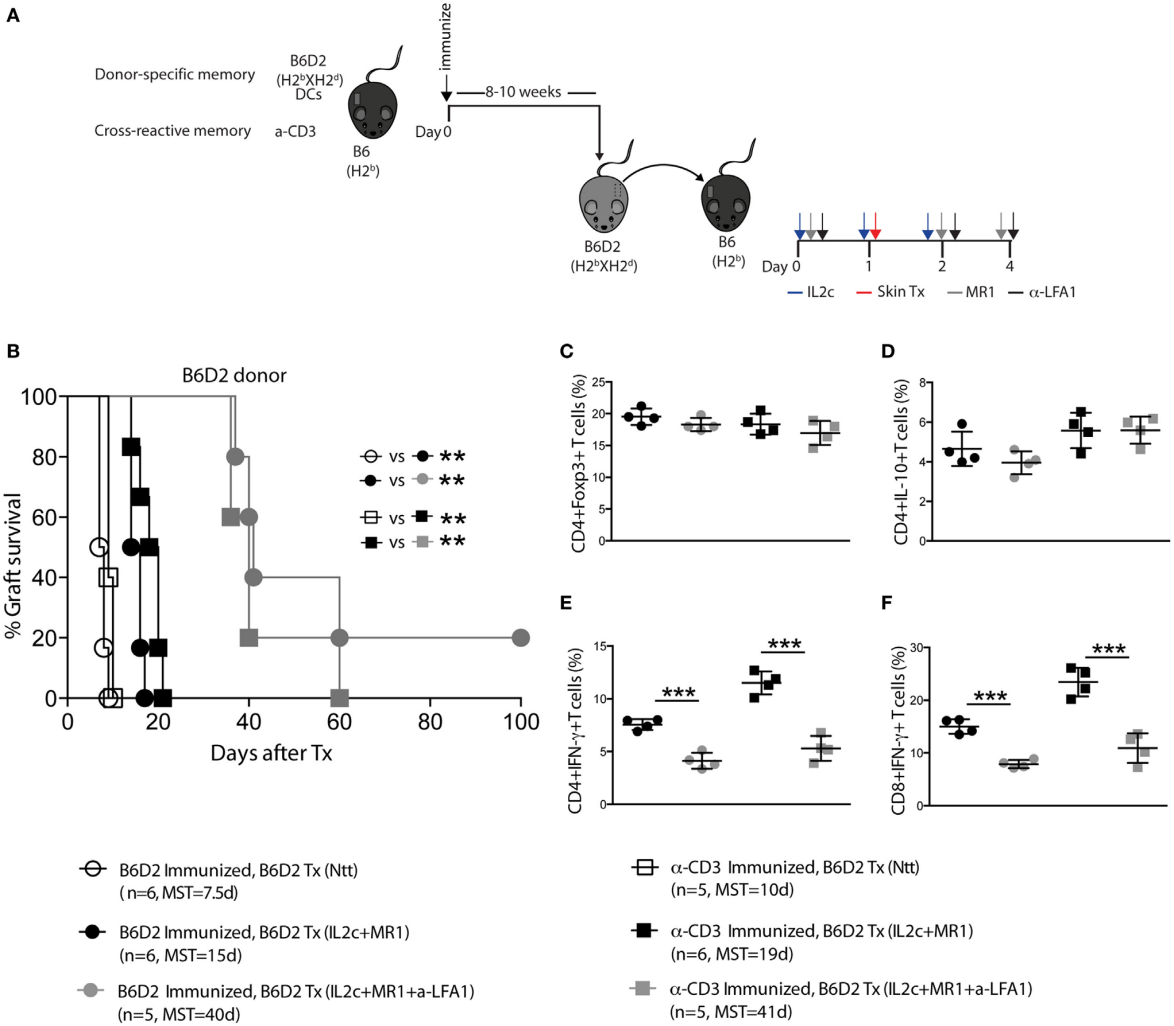
**IL2c in combination with MR1 prolongs MHC-mismatched skin graft survival in the presence of preexisting memory T cells, and this effect is enhanced by anti-LFA-1 treatment**. **(A)** Recipient B6 mice were immunized with either 5 × 10^5^ B6D2 mature dendritic cells (DC) (circles) or 0.5 mg/kg anti-CD3 (squares), followed 8–10 weeks later by a B6D2 skin graft. Mice were left untreated (Ntt) or received IL2c + MR1, with or without anti-LFA-1 (black and gray symbols, respectively). **(B)** Graft survival. **(C–F)** Phenotype and effector function of T cells in the draining lymph nodes at rejection. Frequency of **(C)** CD4^+^Foxp3^+^; **(D)** CD4^+^IL-10^+^; **(E)** CD4^+^IFN-γ^+^, and **(F)** CD8^+^IFN-γ^+^ T cells. *n* = 4–6 mice/group (*P < 0.05, **P < 0.01, and ***P < 0.001).

Alloantigen-specific T- and B-cell sensitization prior to Tx as well as the presence of cross-reactive memory T cells (Tm) have been associated with increased risks of acute and chronic rejection as well as hurdles for tolerance induction (31, 32). Thus, to investigate the clinical relevance of our proposed therapy, we investigated the potential of IL2c + MR1 to promote Tx tolerance in the presence of preexisting donor-specific and cross-reactive memory. B6 mice were either immunized with donor strain DC (alloreactive memory) or with anti-CD3 (cross-reactive memory) before receiving a B6D2 skin graft (Figure 7A). As in naive recipients and using a similar experimental setting, we could demonstrate that IL2c + MR1 therapy allowed selective expansion of antigen-specific CD4^+^Foxp3^+^HY^+^ Treg in the presence of Tm in sensitized mice (data not shown). Taking this finding into account, we hypothesized that IL2c + MR1 given at the time of Tx may still prove efficacious in sensitized mice. In comparison to untreated sensitized recipients that experienced accelerated rejection upon re-exposure to a second graft (MST = 7 and 10 days; in alloreactive and cross-reactive groups, respectively), IL2c + MR1 treatment allowed prolonged graft survival in the presence of both preexisting donor-specific and cross-reactive Tm (MST = 15 and 19 days, respectively) (Figure 7B). However, the presence of Tm before Tx abrogated the induction of tolerance compared to mice with a predominantly naïve immune repertoire.

### The Addition of Anti-LFA-1 Treatment Enhances Allograft Survival in Sensitized Mice

The integrin leukocyte function-associated antigen-1 (LFA-1, CD11a) was shown to be upregulated on Tm and to play a crucial role in the trafficking of these cells to the site of antigenic challenge (33). The addition of anti-LFA-1 to the IL2c + MR1 combination therapy significantly enhanced graft survival in the presence of both preexisting donor-specific and cross-reactive Tm (MST = 40 and 41 days; in alloreactive and cross-reactive groups, respectively) (Figure 7B). The prolonged graft survival in anti-LFA-1-treated mice could not be attributed to augmented immunoregulatory responses as there was no difference in the frequency of CD4^+^Foxp3^+^ and CD4^+^IL-10^+^ T cells in the graft dLN compared to mice that received IL2c + MR1 with no addition of anti-LFA-1 (Figures 7C,D). However, we observed significant decreased IFN-γ production by CD4^+^ and CD8^+^ T cells in anti-LFA-1-treated mice (Figures 7E,F).

**Figure 8:**
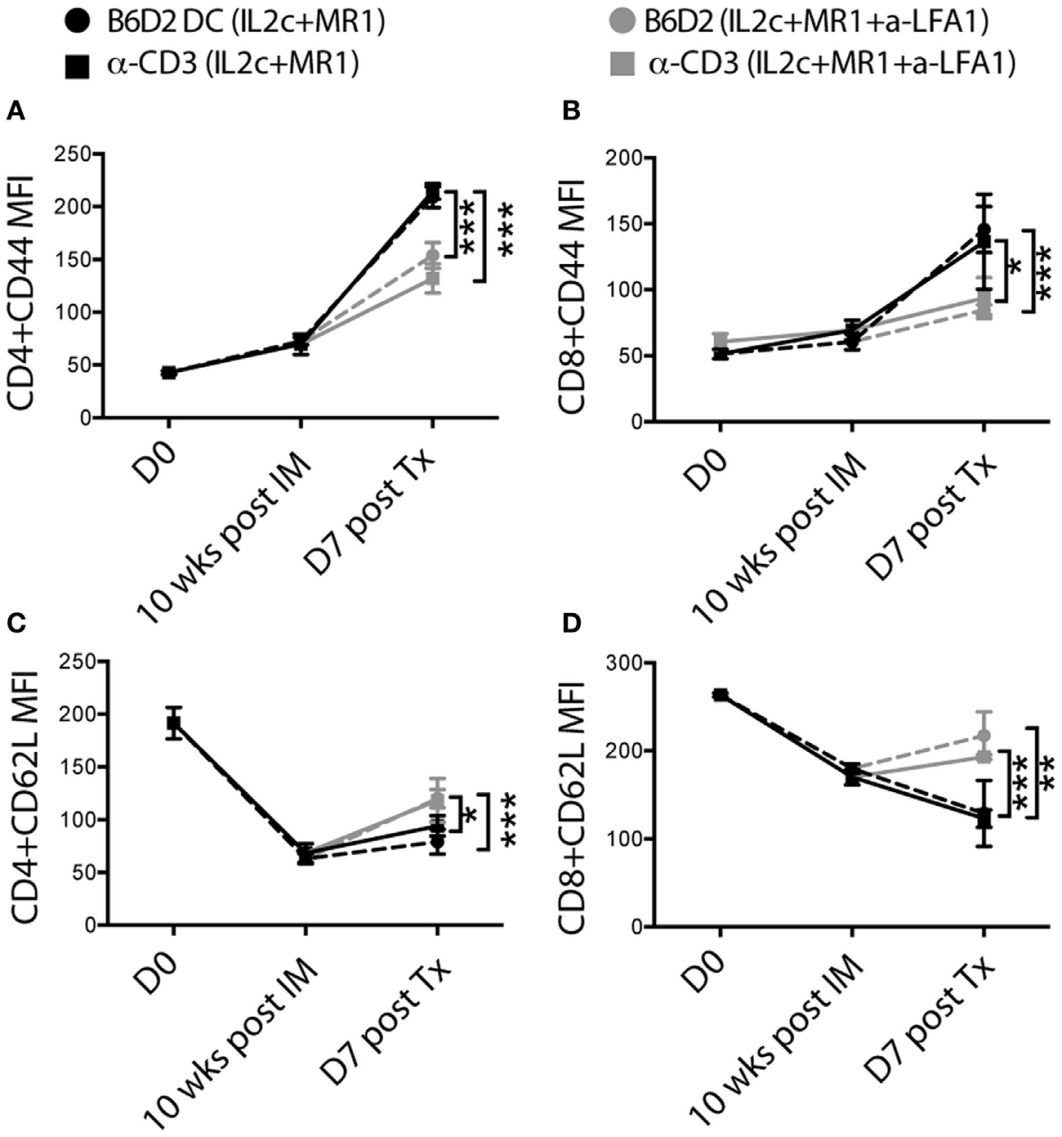
**In pre-sensitized mice, addition of anti-LFA-1 to IL2c-MR1 therapy restricts the activation and effector function of alloreactive T cells in response to an allograft**. B6 mice were tail-bled at day 0, 10 weeks post immunization (with B6D2 DC or anti-CD3), which corresponded to the day of transplantation and at day 7 post B6D2 allograft for flow cytometry analysis. **(A–D)** Changes in mean fluorescence intensity of CD44 and CD62L expression on peripheral CD4^+^ and CD8^+^ T cells. *n* = 4–6 mice/group (*P < 0.05, **P < 0.01, and ***P < 0.001).

To further analyze the added effect of anti-LFA-1 treatment, we compared the kinetics of T-cell activation within experimental groups by monitoring the expression of the activation/memory marker CD44 and of CD62L on peripheral blood T cells at different time-points (8–10 weeks after immunization but prior to Tx, day 7 after Tx, and at rejection). Donor DC pre-sensitized and anti-CD3 pretreated mice that received anti-LFA-1 at the time of Tx, had significantly decreased CD44 and increased CD62L expression on both CD4^+^ and CD8^+^ T cells on day 7 (Figures 8A–D), indicating that the addition of anti-LFA-1 treatment contributed to controlling the activation and homing potential of circulating alloreactive T cells, therefore further prolonging graft survival in combination with IL2c + MR1.

### Donor-Specific B-Cell Responses Mediate Late Rejection in Pre-Sensitized Mice despite the Addition of Anti-LFA-1

**Figure 9:**
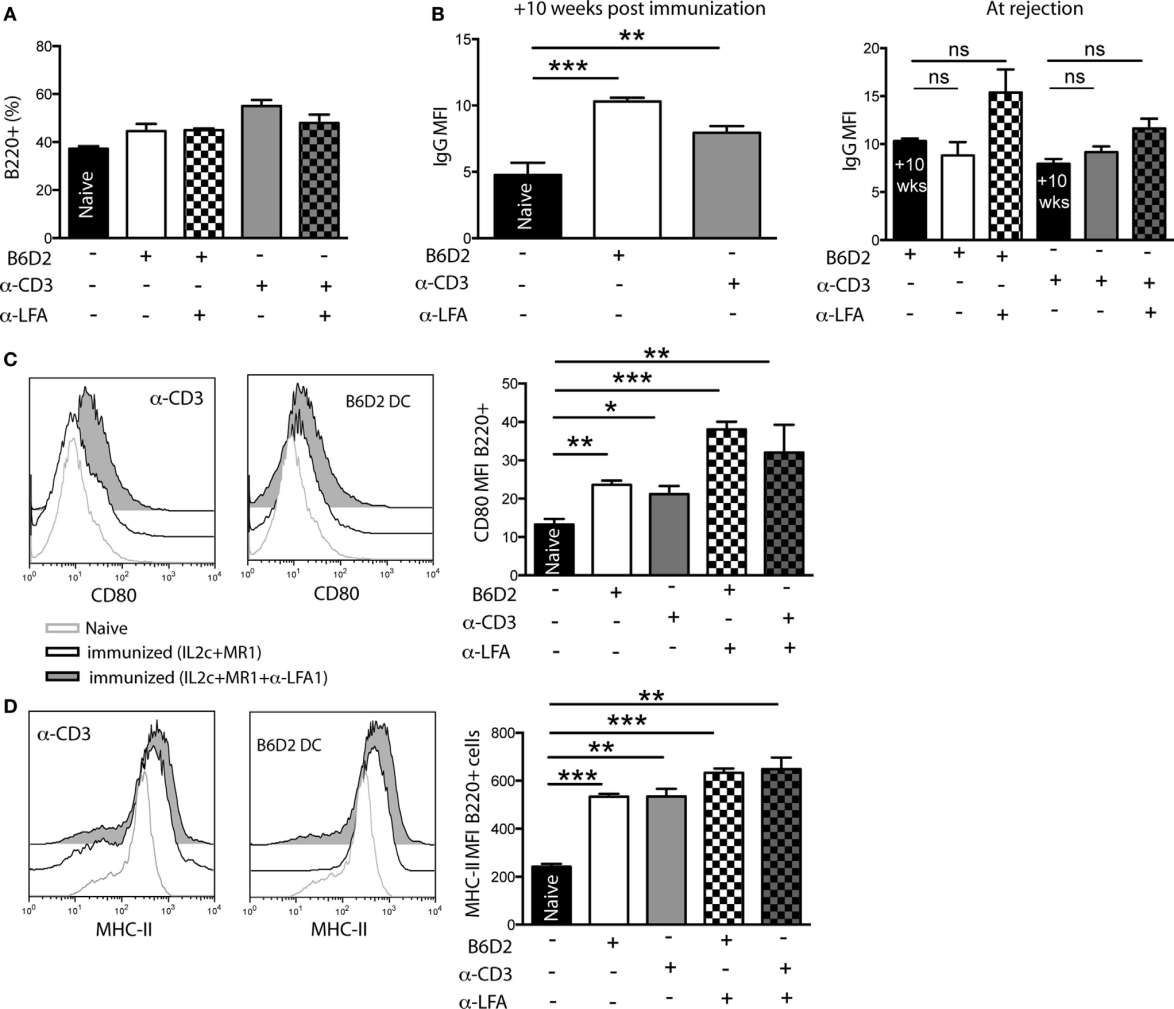
**In pre-sensitized mice, addition of anti-LFA-1 to IL2c-MR1 therapy has no effect on B-cell mediated graft rejection**. B6 mice were immunized [with B6D2 dendritic cells (DC) or anti-CD3] 10 weeks prior to a B6D2 skin graft. All transplanted mice received IL2c-MR1 therapy, without or with anti-LFA-1. At rejection, graft draining lymph nodes (LN) and serum were harvested for flow cytometry analysis. **(A)** Frequency of B220^+^ B cells in graft draining LN. **(B)** Levels of alloantibodies measured at 10 weeks post immunization and at rejection. Mean fluorescence intensity (MFI) of **(C)** CD80 and **(D)** MHC class II expression on B220^+^ B cells in the graft draining LN. *n* = 4–6 mice/group (*P < 0.05, **P < 0.01, and ***P < 0.001).

Recipients that received IL2c + MR1 in combination with anti-LFA-1 eventually underwent late graft rejection, even though immunoregulatory T cells were present and Teff allo responses were dampened. Hence, we analyzed the role of B cells in mediating late rejection in pre-sensitized recipients harboring either donor-specific or cross-reactive memory cells. When analyzing dLN of transplanted mice at rejection, we observed no difference in the frequency of B220^+^ B cells in the dLN of non-immunized compared to sensitized recipients that received IL2c + MR1 alone or with anti-LFA-1 (Figure 9A). However, as measured at the time of graft rejection, treatment with either IL2c + MR1 alone or in combination with anti-LFA-1 did not control the elevated levels of DSA in the serum resulting from sensitization prior to Tx (Figure 9B). We subsequently analyzed the phenotype of B cells present in graft dLN at rejection and observed that neither IL2c + MR1 therapy nor the addition of anti-LFA-1 could control the upregulation of the co-stimulatory molecule CD80 on B cells in pre-sensitized transplanted mice (Figure 9C). Furthermore, in-dependent of the IS treatment, graft dLN B cells had persistent increased expression of MHC class II, which could indicate their in-creased antigen-presenting potential (Figure 9D). These data suggested that, as opposed to the observed regulation of Teff, IL2c + MR1 + anti-LFA-1 therapy had no effect on donor-specific B-cell responses that mediated late rejection in pre-sensitized mice.

## Discussion

In recent years, Treg-based immunotherapy has emerged as a promising strategy to promote operational tolerance after SOT. The possibility to expand *in vivo* Treg using IL-2 would alleviate some hurdles associated with cumbersome and costly *in vitro* procedures, including Treg production in GMP facilities (12, 13, 34, 35). Here, we investigated whether the use of the IL-2/JES6-1 complex (IL2c), designed to specifically expand Treg, would allow the induction of Tx tolerance across MHC barriers, in non-lymphopenic recipients of highly immunogenic skin allografts. We observed that IL2c given at the time of Tx expanded donor-specific Treg that delayed graft rejection, in part by regulating alloreactive T cells activation and effector function. However, IL2c alone was not sufficient to induce Tx tolerance which is consistent with previous studies highlighting the importance of controlling the clonally expanding alloreactive Teff pool to promote Treg-mediated peripheral tolerance (9, 10). In a previous study, we had shown that the adoptive transfer of 2 × 10^6^ donor-specific Treg into non-lymphopenic mice 1 day before an MHC-mismatched skin Tx resulted in similar graft prolongation (MST = 23 days, *P* < 0.001) as described in the current study (5). Thus, in stringent Tx settings, additional immunomodulatory therapies are needed even in the presence of very high Treg frequencies (>10-fold physiological values) at the time of allorecognition. Many experimental models demonstrating the potential of Treg to induce donor-specific Tx tolerance have used lymphopenic mice (7, 8, 25). These models are, however, often biased in favor of Treg (36). Recently, IL2c treatment alone was shown to induce tolerance in immunocompetent recipients of MHC-mismatched pancreatic islets (37). However, we have reported that the streptozotocin-induced diabetes model results in profound transient lymphopenia in peripheral blood and secondary lymphoid organs, resulting in a relative increase in the Treg pool (38). Indeed, we observed a direct cytotoxic effect of streptozotocin, particularly on CD8^+^ T cells and B cells. Thus, it is desirable to perform more studies, using various experimental models and transplanting across different donor-recipient strain combinations, before drawing conclusions on the efficacy of Treg-based immunoregulatory strategies.

The importance of combining Treg-based immunotherapy (either *via* adoptive transfer of high numbers or *in vivo* expansion) with the right IS drugs to promote allograft survival in non-lymphopenic hosts was illustrated in our study. Indeed, while we observed similar IL-2-mediated Treg expansion in combination with Rapa, CTLA-4 Ig, or MR1 (39, 40), only combination with CD154–CD40 co-stimulation blockade promoted tolerance to MHC-mismatched skin grafts. Our data suggest that in our experimental model, while the increased pool of Treg mainly allowed the regulation of CD4^+^ Teff, additional blockade of CD40L prevented efficient priming and differentiation of naïve CD8^+^ T cells (41–43), as well as the activation of alloreactive B cells and the production of DSA. Indeed, CD40 is constitutively expressed on DC and B cells, and its ligation was shown to upregulate the expression of co-stimulatory and MHC molecules, as well as of the transcription factor Blimp-1 in B cells required for the differentiation into plasma cells (44). Thus, the addition of MR1 contributed to the maintenance of allograft tolerance by preventing B-cell activation and the production of deleterious *de novo* DSA (28) as well as possibly limiting chronic indirect pathway activation, which are all important mediators of chronic rejection (45, 46). We also hypothesized that, as compared to CTLA-4 Ig, the effect of MR1 would be even greater in sensitized hosts, as Tm and in particular CD8^+^ Tm were shown to be less dependent on CD28-mediated co-stimulation. Moreover, the administration of CTLA-4 Ig (rather than a CD28 antagonist) may have interfered with the regulatory crosstalk between Treg and APC (47). Finally, although Rapa has been shown to promote Treg survival and reduce alloreactive Teff (48–50), it did not further enhance graft survival when combined with IL2c in our experimental setting.

As supported by flow cytometry phenotypic analysis and *in vitro* suppressive assays, we confirmed that IL2c therapy expanded a subset of Treg and, corroborating recent data, the combination with CD154–CD40 co-stimulation blockade did not hinder their proliferation or suppressive function (51–53). Based on the expression of Helios, we assumed that the expanded Treg collected in the peripheral blood and lymphoid organs were mainly thymic-derived (23). In the light of current data, this implies that IL2c-expanded Treg would be more stable in their Foxp3 expression than peripheral-induced Treg. IL2c treatment did not induce significant expansion of CD8^+^Foxp3^+^ regulatory cells, a subset of potent suppressor cells which has been recently described (Figure S1 in Supplementary Material) (54). Importantly, the expansion of Treg at the time of allorecognition allowed enrichment of donor-specific Treg. Indeed, in the setting of SOT, the efficacy, and superiority (as compared to polyclonal Treg) of donor-specific Treg to induce and maintain tolerance has been highlighted in several studies (5, 7, 25, 55). Antigen-specificity also contributed to specific homing of Treg in the graft dLN and into the grafted tissue to dampen allospecific Teff responses, thus protecting the allograft.

The presence of preexisting donor-reactive memory cells is rarely represented in experimental rodent models of SOT, where the starting pool of T and B cells is almost exclusively naïve, making the induction of tolerance potentially easier to achieve. Therefore, we tested the robustness of IL2c combined with CD154–CD40 co-stimulation blockade to promote MHC-mismatched graft survival in pre-sensitized non-lymphopenic hosts. Our data demonstrated that, as in non-immunized mice, IL2c + MR1 given at the time of Tx allowed the selective *in vivo* expansion of Treg and the induction of IL-10^+^ immunoregulatory CD4^+^ T cells, as well as, to some extent, the control of alloreactive Teff. The addition of anti-LFA-1 further dampened the activation and effector function of alloreactive Teff, hence leading to significant prolonged graft survival. Similarly, in an experimental heart Tx model, anti-LFA-1 treatment inhibited early infiltration of preexisting host CD8^+^ Tm into allografts, leading to prolonged survival (56). In the clinic, anti-LFA-1 (Efalizumab)-based maintenance regimens were shown to promote insulin-independence in type 1 diabetic recipients of pancreatic islets allografts (57, 58). In our experimental setting, while the T-cell alloreactive compartment was controlled by this enhanced combination therapy, late graft loss occurred mainly due to insufficient control of B-cell-mediated alloresponses. Although anti-LFA-1 treatment has been shown to prevent the migration of naïve B cells to T-cell zones within follicular regions of lymphoid organs (59), either it was inefficacious in the presence of preexisting antigen-experienced B cells or possibly supplementary doses should have been administered in our model to prevent *de novo* activation of alloreactive B cells. Moreover, as late rather than early acute rejection occurred in sensitized mice, the antigen-presenting capacity of B cells maintaining chronic indirect pathway T-cell alloresponses, rather than alloantibodies, could also be incriminated in our model and this deserves further investigation (46). Finally, our data also highlight the limited potential of Treg *per se* in regulating B-cell activation after SOT, a finding that may be relevant to translate therapeutic strategies to the clinical setting.

Overall, our data contribute to a better understanding of the complexity of the alloresponse and could help to define new immunomodulatory strategies with a potential to be relevant in the clinic. Although the use of Treg therapy has appeared very promising based on previous experimental data, Treg do not appear to have sufficient potency as a stand-alone therapy in the setting of clinical SOT. Hence, the challenge will be to design an optimized short-term immunomodulatory therapy that could promote Treg homeostasis while targeting the alloreactive naïve and memory T- and B-cell compartments. Based on the present data, we propose to combine the expansion of the donor-specific Treg pool at the time of Tx, together with CD154–CD40 blockade, at least in non-sensitized hosts.

## Ethics Statement

The study was in accordance with Swiss and Canton de Vaud veterinary authorizations (Number 2655.0).

## Author Contributions

LG designed and performed research, analyzed and interpreted data, and wrote the paper. J-CW and RK performed research. MP interpreted data and wrote the paper. DG designed and performed research, analyzed and interpreted data, and wrote the paper.

## Conflict of Interest Statement

The authors declare that the research was conducted in the absence of any commercial or financial relationships that could be construed as a potential conflict of interest.
