# Supplementary material for: IL-2-Mediated In Vivo Expansion of Regulatory T Cells Combined with CD154–CD40 Co-Stimulation Blockade but Not CTLA-4 Ig Prolongs Allograft Survival in Naive and Sensitized Mice
Source: Front Immunol. 2017 Apr 21;8:421. doi: 10.3389/fimmu.2017.00421 (PMC5399033; doi:10.3389/fimmu.2017.00421)
Supplement: Supplementary file 1 [file Data_Sheet_1.DOCX]

Supplementary Material

**IL-2-mediated in vivo expansion of regulatory T cells combined with CD154-CD40 co-stimulation blockade but not CTLA-4 Ig prolongs allograft survival** **in naïve and sensitized mice**

**Authors:** **Lerisa Govender, Jean-Christophe Wyss, Rajesh Kumar, Manuel Pascual, Dela Golshayan^*^**

*** Correspondence:** PD Dr D Golshayan: [Dela.Golshayan@chuv.ch](mailto:Dela.Golshayan@chuv.ch)


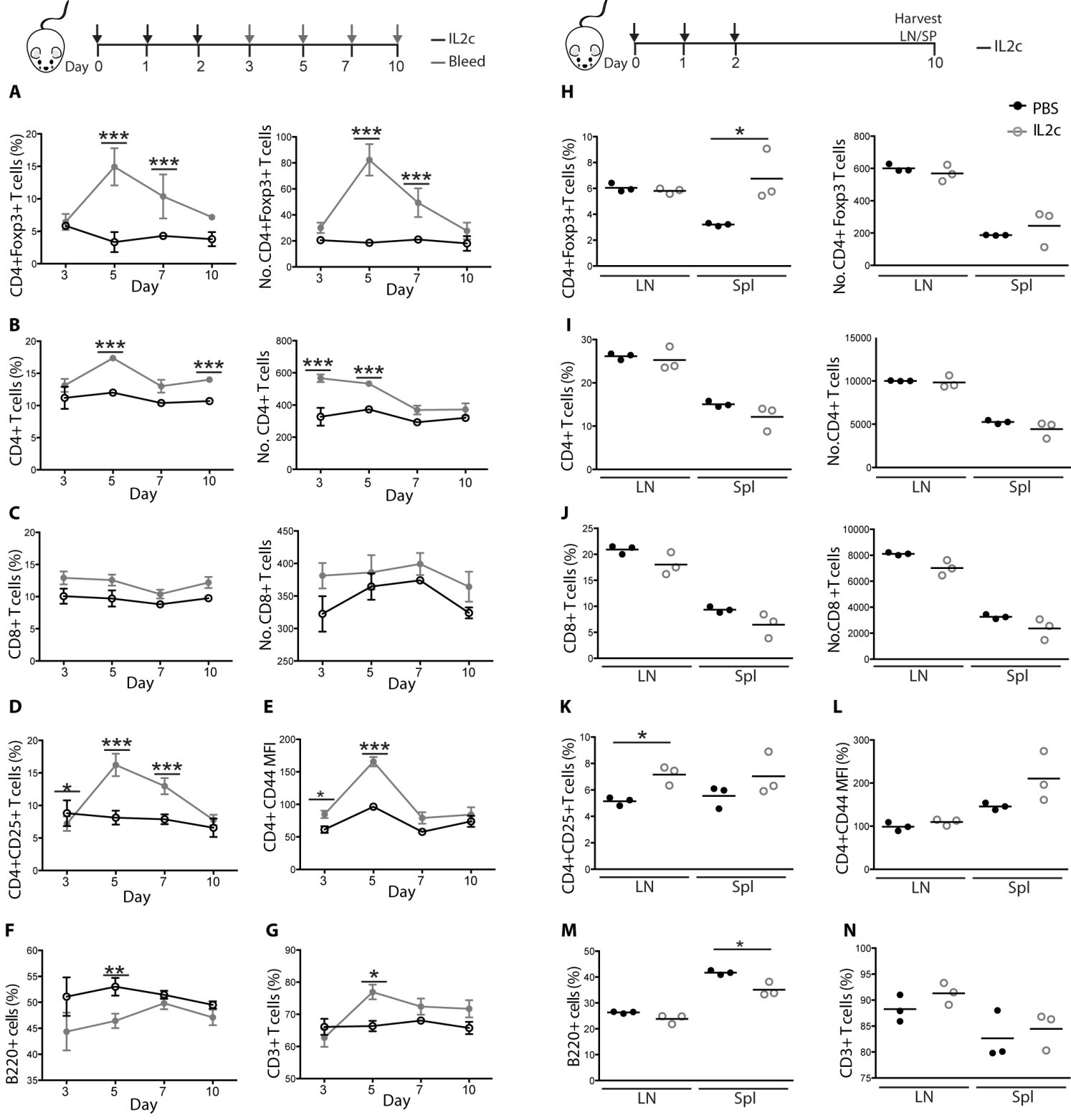


**Supplementary Figure 1. Kinetics and phenotype of immune cells after IL2c treatment.** BALB/c mice were treated with PBS or IL2c and tail-bled on days 3, 5, 7 and 10. Peripheral lymph nodes (LN) and spleen (Spl) were harvested and analyzed on day 10. Frequency and numbers of **A and H.** CD4^+^Foxp3^+^; **B and I.** CD4^+^, **C and J.** CD8^+^ T cells. **D and K.** Frequency of CD4^+^CD25^+^ T cells. **E and L.** MFI of CD44 expression on CD4^+^ T cells. Frequency of **F. and M.** B220^+^ B cells; **G. and N.** CD3^+^ T cells. n=3 mice/group (**P*<0.05, ***P*<0.01, ****P*<0.001).

**
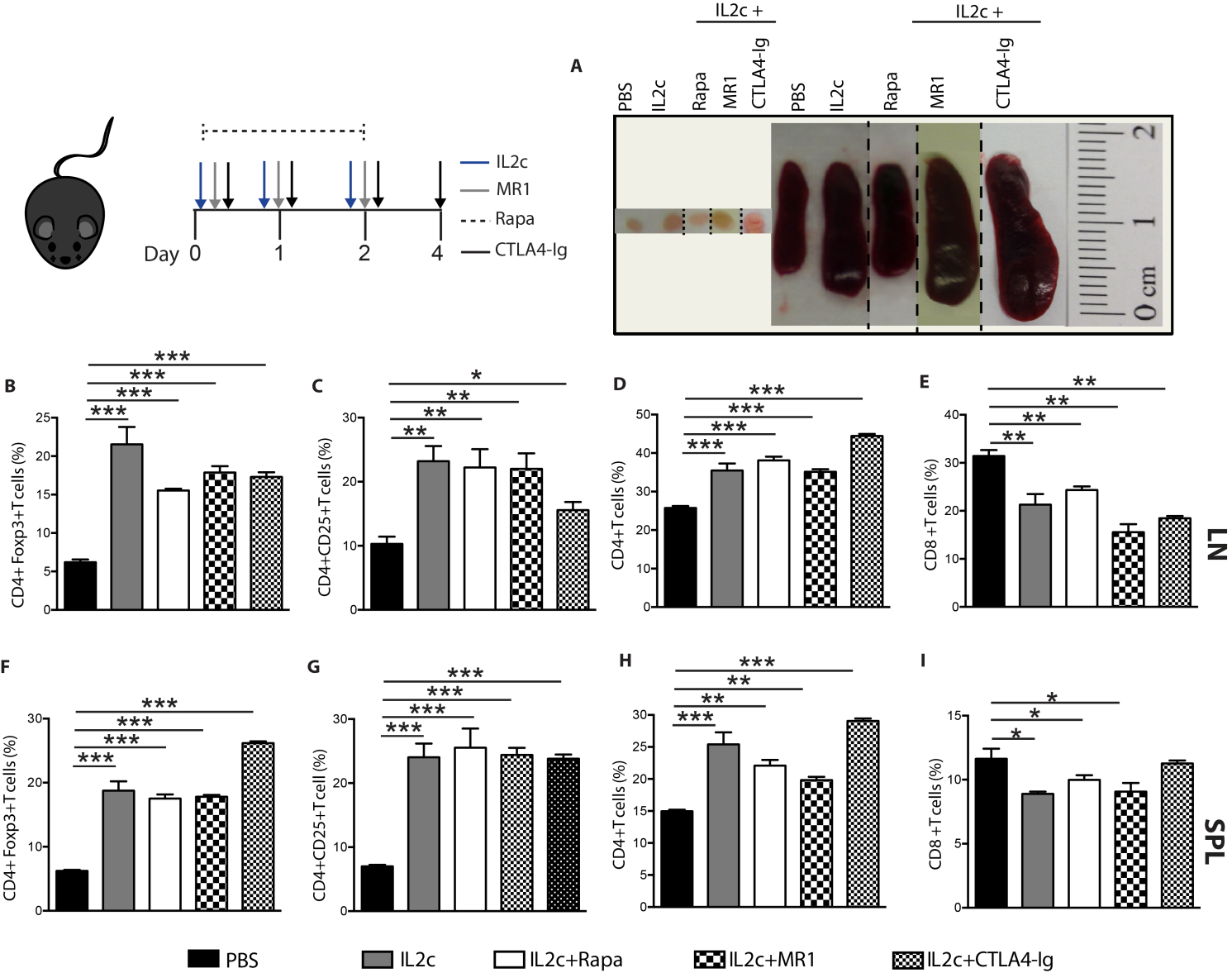
**

**Supplementary Figure 2. IL2c-mediated expansion of Treg *in vivo* is not affected by the presence of rapamycin, MR1 or CTLA-4 Ig.** B6 mice received either PBS, IL2c alone or in combination with Rapa, MR1 or CTLA-4-Ig. On day 5, peripheral lymph nodes (LN) and spleens (SPL) were harvested from each mouse for flow cytometry analysis. **A.** Enlarged lymphoid organs after IL2c treatment. Frequency of **B and F.** CD4^+^Foxp3^+^ T cells; **C and G.** CD4^+^CD25^+^ T cells; **D and H.** CD4^+^ T cells**; E and I.** CD8^+^ T cells in LN and SPL, respectively. n=3-4 mice/group (**P*<0.05, ***P*<0.01, ****P*<0.001).
